# Fear avoidance beliefs as a predictor for long-term sick leave, disability and pain in patients with chronic low back pain

**DOI:** 10.1186/s12891-018-2351-9

**Published:** 2018-12-03

**Authors:** Jeanette Sora Trinderup, Annette Fisker, Carsten Bogh Juhl, Tom Petersen

**Affiliations:** 1 Back Centre Copenhagen, Health Centre Nørrebro, Mimersgade 41, 2200 Copenhagen N, Denmark; 2 CopenRehab, Section of Social Medicine, Department of Public Health, Faculty of Health, University of Copenhagen, Henrik Pontoppidansvej 6, 1st floor, 2200 Copenhagen N, Denmark; 3 Research Unit for Musculoskeletal Function and Physiotherapy, Department of Sports, Science and Clinical Biomechanics, University of Southern Denmark, Campusvej 55, 5230 Odense M, Denmark; 4 Department of Physiotherapy and Occupational Therapy, University Hospital of Copenhagen, Herlev and Gentofte, Kildegårdsvej 28, 2900 Hellerup, Denmark

**Keywords:** Chronic low back pain, Fear avoidance beliefs, Fear Avoidance Beliefs Questionnaire, Sick leave, Disability, Prognostic factors

## Abstract

**Background:**

Subgrouping patients with chronic low back pain is recommended prior to selecting treatment strategy, and fear avoidance beliefs is a commonly addressed psychological factor used to help this subgrouping. The results of the predictive value of fear avoidance beliefs in patients with chronic low back pain in prognostic studies are, however, not in concordance. Therefore, the objective of this study was to examine the association between fear avoidance beliefs at baseline and unsuccessful outcome on sick leave, disability and pain at 12-month follow-up in patients with entirely chronic low back pain.

**Methods:**

A secondary analysis of data from a randomised controlled trial. Patients with chronic low back pain (*n* = 559) completed questionnaires at baseline and after 12 months. Multiple logistic regression analyses were conducted to examine the association between fear avoidance beliefs and the outcomes sick leave, disability and pain.

**Results:**

Higher fear avoidance beliefs about work at baseline were found to be significantly associated with still being on sick leave (OR 1.11; 95% CI 1.02–1.20) and having no reduction in pain (OR 1.04; 95% CI 1.01–1.08) after 12 months and may be associated with having no reduction in disability (OR 1.03; 95% CI 1.00–1.06) after 12 months (lower limit of 95% CI close to 1.00). Fear avoidance beliefs about physical activity were not found to be associated with the three outcomes.

**Conclusions:**

High fear avoidance beliefs about work are associated with continuous sick leave after 1 year in patients with chronic low back pain. This finding might assist clinicians in choosing targeted treatment strategies in subgroups of working patients with chronic low back pain.

**Electronic supplementary material:**

The online version of this article (10.1186/s12891-018-2351-9) contains supplementary material, which is available to authorized users.

## Background

In high-income countries it is estimated that 2–5% of the population have chronic low back pain (CLBP); i.e. low back pain (LBP) for at least 3 months [1]. Despite the large amount of research conducted in the field of CLBP, the treatment effect is moderate at its best [2, 3]. The heterogeneity of patients with CLBP indicates that it might be beneficial to classify the patients into subgroups prior to selecting treatment strategy, as subgroups with various characteristics respond differently to the same treatment [4]. There is still a lack of evidence with respect to clinical relevant subgroups of patients with CLBP [5].

Screening for psychosocial prognostic factors is recommended in the process of subgrouping patients with CLBP [5]. One of the psychological factors most commonly assessed in prognostic studies on CLBP is fear avoidance beliefs [6, 7]. Patients with fear of pain will be at risk of developing avoidance behaviour as a means to reduce pain, which might lead to reduced physical activity, increased disability and absence from work [8, 9]. Although the predictive value of fear avoidance beliefs is commonly assessed in prognostic studies, the results are not in concordance and the existing studies contain a mix of patients with LBP and CLBP [6, 10].

The objective of this study was to examine the association between fear avoidance beliefs at baseline and the outcomes sick leave, disability and pain at 12-month follow-up in patients with CLBP.

## Methods

### Study design

This study was secondary analysis of data from a randomised controlled trial [11] with a 12-month follow-up. The overall aim of the randomised controlled trial was to evaluate the effectiveness of a work-orientated multidisciplinary intervention coordinated by a physiotherapist. The control group received usual multidisciplinary care by a team of a physiotherapist, chiropractor, rheumatologist and social worker. The intervention group received a work-oriented multidisciplinary intervention consisting of the aforementioned professionals plus a psychologist, occupational physician, occupational therapist and a case manager from the municipal sickness benefit office. Data was collected by questionnaires during the period from September 2009 to December 2013 at an outpatient back care centre in Copenhagen. Details of the randomised controlled trial have been published elsewhere [11].

### Patients

After approval by the Danish Regional Ethics Committee, The Capital Region of Denmark (File number H-C-2008-112), patients living in the municipality of Copenhagen, Denmark, were referred from general practitioner, rheumatologist or municipal sickness benefit office for treatment of persistent LBP. All patients received oral and written information about the randomised controlled trial and gave written informed consent prior to participation.

The inclusion criteria were working age adults (18–65 years) with LBP for at least 3 months, on sick leave or at risk for eminent sick leave. Exclusion criteria were pending application for early retirement pension, pregnancy, comorbidity (i.e. severe consequences of cancer, cardiopulmonary diseases, mental or psychological diseases) or difficulties in reading and writing Danish.

### Measurements

All patients completed baseline questionnaires including the following variables: age, sex, Body Mass Index, education (years after primary school), smoking, alcohol intake, leisure physical activity level, sick leave due to LBP, duration of sick leave, job status, current compensation case, physical job demands, general health status measured on the Short Form 36 [12], anxiety and depression measured on the Symptom Checklist-90-Revised [13], pain intensity measured on the Numeric Pain Rating Scale [14], disability measured on the 23-item modified Roland Morris Disability Questionnaire [15, 16] and fear avoidance beliefs measured on the Fear Avoidance Beliefs Questionnaire (FABQ) [8]. In addition, treatment group during the 12-week intervention in the randomised controlled trial was recorded.

### Statistical analysis

The outcome variable sick leave was defined as unsuccessful, if a patient on sick leave at baseline was still on sick leave at 12-month follow-up. The modified Roland Morris Disability Questionnaire and the Numeric Pain Rating Scale were dichotomized according to recommended scores of minimal important change [17], as this enabled comparison with previous studies. The outcome variables disability and pain were defined as unsuccessful, if a patient had a reduction of less than 5 points on the modified Roland Morris Disability Questionnaire and less than 6 points on the Numeric Pain Rating Scale at 12-month follow-up. In case of missing values on outcome variables, dropout analyses were performed.

Multiple logistic regression analyses were used to examine the association between fear avoidance beliefs and the outcomes sick leave, disability and pain. The following variables were included as confounders à priori on the basis of known or presumed risk factors between fear avoidance beliefs and the three outcomes: sex, age, pain intensity [18], disability [19] and depression [20]. Additionally, physical job demands [21] has been reported to be a risk factor for the outcome sick leave.

The analyses were conducted separately for each outcome in five steps: first, test for the assumption of normal distributions of the residuals; second, univariate analyses between the outcome variables and the independent variables to compute crude estimates; third, test for collinearity between the continuous variables based on Pearson’s *r* > 0.5; fourth, multiple logistic regression analyses were conducted using backward stepwise elimination (until none of the variables had *p*-value > 0.1) in order to identify the best fitted model with the highest explanatory value (R^2^); finally, tests for interaction were performed to examine whether any interaction joint increased the model fit, evaluated on Wald tests.

The following sensitivity analyses were performed: analysis including the variables with a *p*-value below 0.2 in the univariate analysis; analysis with fear avoidance beliefs about work (low, 0–29; high, 30–42) and fear avoidance beliefs about physical activity (low, 0–14; high, 15–24) as dichotomous variables [22]; finally, a simpler multiple logistic regression analysis, including only the two fear avoidance beliefs subscales and the variables found significant in the final model.

The outcomes were reported in odds ratios (OR) with 95% confidence intervals (CI). A *p*-value below 0.05 was considered statistical significant. Data were analysed using the statistical package STATA/IC 14.1 for Mac (Stata Corporation, College Station, TX, USA).

## Results

A total of 559 patients were included in the study. The mean age of the patients was 38.9 years (SD 10.4) and 47.1% were women. Median duration of LBP was 11 months (IQR 5–33). Two hundred seventy-three patients (51.4%) had a duration of LBP between 3 to 12 months and 275 patients (49.8%) were on sick leave at baseline (Table 1). The proportions of missing values on the outcome variables at 12-month follow-up was 41% for sick leave, 34% for disability and 35% for pain (Table 2).

**Table 1:** Baseline characteristics of the total sample of patients and those on sick leave

Variable	Total sample (*n* = 559)	Number of responders	Subgroup on sick leave at baseline (*n* = 275)	Number of Responders
Sex, female, *n* (%)	263 (47.05)	559	114 (41.45)	275
Age, years, mean (SD)	38.90 (10.42)	559	38.68 (10.75)	275
Body Mass Index, mean (SD)	25.53 (4.47)	543	25.05 (4.63)	267
Education after primary school, *n* (%)		548		268
<2 years	123 (22.45)		83 (30.97)	
2–4 years	370 (67.52)		167 (62.31)	
>4 years	32 (5.84)		6 (2.24)	
Other	23 (4.20)		12 (4.48)	
Current smoker, no, *n* (%)	299 (54.07)	553	123 (45.56)	270
Alcohol, ≤7 units/week, *n* (%)	412 (76.40)	540	202 (75.94)	266
Physical activity level leisure, *n* (%)		547		268
Little-some	424 (77.51)		225 (83.96)	
Moderate-high	123 (22.49)		43 (16.04)	
Sick leave, yes, *n* (%)	275 (49.82)	552	275 (100)	268
Duration of sick leave, weeks, mean (SD)	12.45 (15.81)	261	12.47 (15.87)	258
Employment, no, *n* (%)	127 (23.09)	550	97 (35.93)	270
Compensation case, yes, *n* (%)	87 (16.35)	532	52 (20.00)	260
Physical job demands, *n* (%)		542		263
None	163 (30.07)		61(23.19)	
Little	78 (14.39)		36 (13.69)	
Some	198 (36.53)		97 (36.88)	
Heavy	103 (19.00)		69 (26.34)	
Physical health, 0–100, mean (SD)	50.74 (8.45)	495	49.11 (8.03)	237
Mental health, 0–100, mean (SD)	49.92 (10.32)	495	47.91 (10.26)	237
Depression, 0–4, mean (SD)	1.10 (0.84)	544	1.23 (0.86)	267
Anxiety, 0–4, mean (SD)	0.68 (0.66)	536	0.76 (0.67)	263
Duration of LBP^a^, <12 months, *n* (%)	273 (51.41)	531	148 (56.27)	263
Pain intensity^b^, 0–30, mean (SD)	17.66 (5.66)	559	18.30 (5.62)	271
Age at first episode of LBP^b^, years, mean (SD)	27.79 (11.74)	542	28.87 (11.79)	261
Family history of LBP^a^, yes, *n* (%)	234 (42.70)	548	118 (45.70)	270
Disability, 0–23, mean (SD)	13.75 (4.94)	559	14.62 (4.64)	275
FAB work^c^, 0–42, mean (SD)	24.31 (11.34)	514	29.55 (9.63)	245
FAB physical activity^d^, 0–24, mean (SD)	15.46 (5.34)	522	16.41 (3.33)	249
Group, intervention, *n* (%)	298 (53.31)	559	150 (54.55)	275

*SD* standard deviation

^a^Low back pain

^b^The back pain questionnaire included 3 separate 11-point numeric rating scales (0–30) comprising the following items: pain at the moment, the worst pain within the past 2 weeks, and the average level of pain within the last 2 weeks. These summed to a total score ranging from 0 points (no back pain at all) to 30 points (worst possible back pain)

^c^Fear avoidance beliefs about work

^d^Fear avoidance beliefs about physical activity

**Table 2:** Number of patients included in the outcome variables sick leave, disability and pain

Outcome variable	Successful (*n*)	Unsuccessful (*n*)	Missing values (*n*)	Total (*n*)
Sick leave^a^	121	40	114	275
Disability^b^	169	200	190	559
Pain^c^	172	191	196	559

^a^Successful if no longer on sick leave, unsuccessful if still on sick leave at 12-month follow-up

^b^Successful if reduction ≥5 on the modified Roland Morris Disability Questionnaire, unsuccessful if <5 points reduction at 12-month follow-up

^c^Successful if reduction ≥6 on the Numeric Pain Rating Scale, unsuccessful if <6 point reduction at 12-month follow-up

For the outcome variable sick leave, the dropout-patients differed significantly from the patients included in the analyses by having lower age and earlier debut of their first episode of LBP at baseline (Additional file 1: Table S1). For the outcome variable disability, the dropout-patients differed significantly from the patients included in the analyses by having lower age and earlier debut of their first episode of LBP, more were male, had higher Body Mass Index, lower education, more were on sick leave and fewer patients were in the intervention group at baseline (Additional file 2: Table S2). For the outcome pain, the dropout-patients differed significantly from the patients included in the analyses by having lower age, earlier debut of their first episode of LBP, higher Body Mass Index, lower education and fewer patients were in the intervention group at baseline (Additional file 3: Table S3). The results of the univariate analysis with respect to successful outcome are presented in Table 3.

**Table 3:** Univariate analyses of the association between baseline variables and successful outcome on sick leave, disability and pain

Variable	Sick leave (*n* = 161)			Disability (*n* = 369)			Pain (*n* = 363)		
	OR	95% CI	*n*	OR	95% CI	*n*	OR	95% CI	*n*
Sex, female	1.17	0.57–2.40	161	1.18	0.78–1.77	369	1.80*	1.19–2.73	363
Age, years	0.98	0.95–1.01	161	1.00	0.98–1.02	369	0.99	0.97–1.01	363
Body Mass Index	0.30^L^	0.03–2.91	157	0.81 ^L^	0.22–2.96	359	0.33 ^L^ ⋄	0.09–1.25	353
Education after primary school			159			363			357
<2 years	1.00			1.00			1.00		
2–4 years	1.00	0.43–2.32		1.44⋄	0.84–2.48		1.03	0.60–1.78	
>4 years	0.91	0.08–9.74		2.40⋄	0.80–1.16		1.46	0.51–4.17	
Other	0.38	0.09–1.69		1.71	0.58–5.05		0.91	0.30–2.73	
Current smoker, no	2.26^*^	1.06–4.80	159	1.44♦	0.95–2.19	365	1.72*	1.13–2.63	359
Alcohol, >7 units/week	1.18	0.48–2.86	155	0.79	0.49–1.28	360	1.24	0.76–2.03	355
Physical activity level leisure, little-some	1.07	0.42–2.77	157	0.85	0.52–1.38	362	1.30	0.80–2.12	356
Sick leave, yes	–	–	161	1.14	0.75–1.72	365	1.27	0.83–1.92	259
Duration of sick leave, weeks	0.67 ^L^ ♦	0.45–1.01	150						
Employment, no	0.49♦	0.23–1.05	157	0.58*	0.35–0.98	363	0.92	0.56–1.53	357
Compensation case, no	1.52	0.63–3.71	153	1.17	0.66–2.09	350	1.91*	1.04–3.49	345
Physical job demands			156			360			355
None	1.00			1.00			1.00		
Little	1.13	0.30–4.33		0.53♦	0.28–1.02		0.71	0.37–1.36	
Some	0.84	0.32–2.21		1.03	0.62–1.71		1.01	0.61–1.67	
Heavy	0.58	0.20–1.67		0.62⋄	0.33–1.17		0.34**	0.17–0.68	
Physical health, 0–100	1.01∅	0.96–1.06	142	0.99∅	0.96–1.01	327	0.99∅	0.96–1.01	322
Mental health, 0–100	1.04♦∅	0.99–1.08	142	0.99∅	0.97–1.00	327	1.00∅	0.98–1.02	322
Depression, 0–4	0.86	0.56–1.34	158	1.04 ^L^	0.82–1.32	344	1.02 ^L^	0.80–1.29	339
Anxiety, 0–4	2.34 ^L^ ∅	0.59–3.87	141	1.03 ^L^ ∅	0.81–1.32	318	0.95 ^L^ ∅	0.74–1.22	313
Duration low back pain, ≥12 months	0.83	0.39–1.76	152	0.36**	0.23–0.55	350	0.67♦	0.44–1.02	346
Pain intensity, 0–30	0.97	0.91–1.03	159	0.97♦	0.93–1.00	366	1.10**	1.05–1.14	363
Age at first episode of low back pain, years	0.98⋄∅	0.95–1.01	150	1.23 ^L^ ∅	0.76–2.00	355	1.02 _L_ ∅	0.63–1.66	349
Family history of low back pain, no	1.26	0.61–2.63	158	1.11	0.73–1.69	363	1.16	0.76–1.77	357
Disability, 0–23	0.99	0.91–1.07	161	1.08**	1.04–1.13	369	1.03	0.99–1.07	363
Fear avoidance beliefs work, 0–42	0.93**	0.88–0.98	145	0.98♦	0.96–1.00	341	0.98*	0.96–0.99	335
Fear avoidance beliefs physical activity, 0–24	0.97	0.90–1.04	149	1.00	0.96–1.04	346	0.98	0.94–1.02	340
Group, intervention	0.98	0.48–2.02	161	1.15	0.76–1.74	369	1.64*	1.08–2.50	363

*OR* Odds ratio, *CI* confidence intervals, ^L^Logarithmic transformed, *p-value < 0.05, **p-value < 0.01, ♦*p*-value < 0.1, ⋄*p*-value < 0.2, ∅removed due to collinearity

In the final adjusted analyses, higher fear avoidance beliefs about work (adjusted OR 1.11 per increased score of one point; 95% CI 1.02 to 1.20) and being a smoker (adjusted OR 3.17; 95% CI 1.02 to 9.87) at baseline were significantly associated with unsuccessful outcome on sick leave (Table 4).

**Table 4:** Final model of the associations between fear avoidance beliefs at baseline and unsuccessful outcome at 12-month follow-up

Variable	Sick leave (*n* = 113)		Disability (*n* = 286)		Pain (*n* = 284)	
	OR	95% CI	OR	95% CI	OR	95% CI
High fear avoidance beliefs about work, 0–42	1.11*	1.02–1.20	1.03*	1.00–1.06	1.04*	1.01–1.08
Smoking	3.17*	1.02–9.87	1.75*	1.01–3.01		
High pain intensity, 0–30			1.10**	1.06–1.16		
Low pain intensity, 0–30					1.14**	1.08–1.20
Low disability (log), 0–23			1.16**	1.08–1.25		
Duration of low back pain ≥12 months and little physical job demands^a^			4.35*	1.22–14.29		
Male and little physical job demands^a^					4.00*	1.06–16.67

*OR* Odds ratio, *CI* confidence intervals, *log* logarithmic transformed, *p-value < 0.05, **p-value < 0.01, ^a^interaction

The outcome sick leave adjusted for age, sex, pain intensity, disability, depression, duration of sick leave (log) and the interaction joint physical job demands, employment and sex scores at baseline. The outcome disability adjusted for age, sex, depression (log), smoking, pain intensity, disability and the interaction joint duration of low back pain and physical job demands scores at baseline. The outcome pain adjusted for age, sex, disability, depression (log), duration of low back pain, smoking and the interaction joint sex and physical job demands scores at baseline

Analysing unsuccessful outcome on disability, higher fear avoidance beliefs about work showed a slight association (adjusted OR 1.03 per increased score of one point; 95% CI 1.00 to 1.06), but the lower limit of the 95% CI was close to 1.00. Significant associations were found between higher pain intensity (adjusted OR 1.10; 95% CI 1.06 to 1.16), lower disability (adjusted OR 1.16; 95% CI 1.08 to 1.25), being a smoker (adjusted OR 1.75; 95% CI 1.01 to 3.01) and having LBP for more than 12 months combined with little physical job demands (adjusted OR 4.35; 95% CI 1.22 to 14.29) at baseline and unsuccessful outcome on disability at 12-month follow-up (Table 4). The final model included a significant interaction between LBP duration and physical job demands, as the inclusion of this interaction joint improved the model slightly with an increased R^2^ from 0.08 to 0.09.

Higher fear avoidance beliefs about work (adjusted OR 1.04 per increased score of one point; 95% CI 1.01 to 1.08), lower pain intensity (adjusted OR 1.14; 95% CI 1.08 to 1.20) and the combination of being male with little physical job demands (adjusted OR 4.00; 95% CI 1.06 to 16.67) at baseline were significantly associated with unsuccessful outcome on pain at 12-month follow-up (Table 4). The final model included a significant interaction between sex and physical job demands, as the inclusion of this interaction joint improved the model slightly with an increased R^2^ from 0.14 to 0.15.

None of the sensitivity analyses changed the results markedly (Additional file 4: Table S4).

## Discussion

The main findings of this study were that high fear avoidance beliefs about work at baseline were significantly associated with still being on sick leave, having no improvement in disability and no improvement in pain after 1 year in patients with CLBP. To our knowledge, this is the first study to investigate the association between fear avoidance beliefs, measured on the FABQ work and the FABQ physical activity separately at baseline and the outcomes sick leave, disability and pain after 1 year in a large sample of patients with entirely CLBP.

The findings of this study are supported by the existing literature regarding sick leave in a systematic review by Wertli et al. [6], in which two studies reported that higher levels of fear avoidance beliefs were related to lower chances returning to work in patients with CLBP [23, 24]. A direct comparison of these two studies to the present study is, however, not possible, since the former studies used the FABQ total score, whereas the present study analysed on the FABQ work and the FABQ physical activity subscales separately. The associations between fear avoidance beliefs about work and the outcomes disability and pain in patients with CLBP have been found in a few previous studies [25, 26]. Overall, the results suggest that fear avoidance beliefs about work are more strongly associated with the outcome sick leave than with the outcomes disability and pain in patients with CLBP. Furthermore, the association between fear avoidance beliefs about work and disability found in our study may be uncertain in as much as the lower limit of the 95% confidence interval is close to 1.00. This might not be surprising, since the FABQ work subscale was developed specifically to measure fear avoidance beliefs about work in relation to work loss [8]. This study included patients at sick leave as well as patients at risk for transitioning to sick leave. The 50% patients at sick leave are expected to have had relatively higher scores on the FABQ at baseline and might have contributed the most to the predictive value of fear avoidance beliefs about work found in this study. The finding that fear avoidance beliefs about physical activity were not associated with any of the examined outcomes is in accordance with those of previous studies [27, 28]. It has been suggested that the two subscales of the FABQ measure the same construct, namely pain-related fear [8], in which case both subscales would have been expected to be associated with the examined outcomes. However, the non-significant associations between fear avoidance beliefs about physical activity and the outcomes in our studies might indicate that fear avoidance beliefs about physical activity reflect other domains, i.e. lack of motivation or poor expectations regarding recovery, as previously stated [29].

In the present study, the levels of pain intensity and disability at baseline were associated with unsuccessful outcome with respect to disability and pain. Higher pain intensity was related to unsuccessful outcome in disability scores, whereas lower pain intensity was related to unsuccessful outcome in pain scores. A possible explanation for the opposing impact of pain intensity may be that patients with a high baseline pain level might have a better chance of reducing pain compared to patients with lower baseline pain levels. These findings regarding disability and pain were in line with the results of previous studies [18, 30, 31], but not in concurrence with a systematic review reporting no association with disability [32].

Although the variables in the final models were significantly associated with the three outcomes, the relatively low R^2^s indicate that neither of the models appear to offer a full explanation of the outcomes examined.

### Limitations and strengths

The main limitation in the present study is the risk of selection bias due to missing values on the outcome variables. The proportions of missing values of 41, 34 and 35% in the outcome variables sick leave, disability and pain, respectively, might have caused misleading results inasmuch as the patients included in the analyses differed significantly from the dropouts in several characteristics. In our opinion, the relatively large amount of dropouts on the outcome variables were not likely to cause an over- or underestimation of the associations, since none of these variables were significantly associated with the outcomes in the univariate analyses (Table 3), and the differences between the patients included in the analyses and the dropouts were minimal (Additional files 1, 2 and 3: Tables S1–S3).

Another limitation in the present study is missing values on the outcome variables. In this study, although none of the variables included in the multiple logistic regression analyses had more than 10% missing values (Table 3), the final number of observations included in the analyses of the outcomes sick leave, disability and pain were reduced from a sample size of 161 to 113, 302 to 286, and 363 to 284, respectively. However, the results of the simpler multiple logistic regression analyses did not change the ORs for the association between fear avoidance beliefs about work and the outcomes disability and pain, and the OR for the outcome sick leave decreased only slightly (Additional file 4: Table S4). This might indicate that the results are relatively robust.

This study was conducted as a secondary analysis of a randomised controlled trial. Consequently, information on factors considered important for the outcomes may have been missed, i.e. catastrophizing and job satisfaction [33–35]. Furthermore, using data from an intervention study holds the risk of the intervention confounding the associations. However, inasmuch as the variable “group” was not significantly associated with the outcomes in any of the adjusted analyses, this aspect is not likely to be a serious risk in the present study. We did not include treatment group interactions in the model because no difference was found between groups in the original randomised controlled trial [11]. It would have been of interest to report the number of patients that transitioned to sick leave during the 12-month follow-up. Unfortunately, data for estimating this number and performing separate analysis on how these patients fared are not available.

It is a strength in the present study that both à priori selected variables and variables with a *p*-value below 0.1 were included in the adjusted analyses. Including à priori variables that are known or presumed to be risk factors between fear avoidance beliefs and the outcomes of interest can increase the comparability to results from previous studies and might prevent the results from being too closely fitted to the data set [36]. Had the multiple logistic regression analyses been conducted solely based on the variables with a *p*-value below 0.1, the backward stepwise elimination may have resulted in an over-optimistic model and random chance associations with the outcomes [37].

### Clinical implications and further research

In summary, the findings of this study indicate that higher fear avoidance beliefs about work at baseline are associated with unsuccessful outcome with respect to sick leave, disability and pain in patients with CLBP after 1 year. Given the inconsistency in the existing literature, more studies are needed prior to making any firm recommendations for the use of the FABQ in clinical practice.

It is unlikely that fear avoidance beliefs are a stand-alone predictor of long-term sick leave, disability and pain in patients with CLBP. Therefore, findings on the FABQ may be included as a part of a more comprehensive composite classification in combination with other questionnaires known to be of value in a treatment oriented subgrouping of patients with CLBP, e.g. the STarT Back Screening Tool [38]. This questionnaire has been validated as a prognostic screening method to allocate patients with mixed duration of LBP into low, medium or high risk subgroups [38]. The use of the FABQ to provide further specific information on patients in the high risk subgroup might help clinicians to better understand the clinical course of patients with CLBP and to identify the individual predictors that need to be targeted in the treatment strategy.

## Conclusion

The results of this study indicate that high fear avoidance beliefs about work may help the identification of patients with CLBP who are at risk of continuous sick leave after 1 year. The magnitude of the associations between fear avoidance beliefs about work and disability and pain were small and future studies investigating these associations in a subgroup of patients with CLBP are warranted until firm conclusions can be made.

## Additional files

Additional file 1: **Table S1.** Dropout analysis comparing baseline characteristics of patients included in the analysis of sick leave to those not included. (PDF 63 kb)

Additional file 2: **Table S2.** Dropout analysis comparing baseline characteristics of patients included in the analysis of disability to those not included. (PDF 62 kb)

Additional file 3: **Table S3.** Dropout analysis comparing baseline characteristics of patients included in the analysis of pain to those not included. (PDF 64 kb)

Additional file 4: **Table S4.** Simpler model of association between fear avoidance beliefs at baseline and unsuccessful outcome at 12-month follow-up. (PDF 53 kb)

## Abbreviations

CI: Confidence Intervals
CLBP: Chronic low back pain
FABQ: Fear Avoidance Beliefs Questionnaire
IQR: Interquartile range
LBP: Low back pain
OR: Odds Ratio
SD: Standard deviation
