# Supplementary material for: Fear avoidance beliefs as a predictor for long-term sick leave, disability and pain in patients with chronic low back pain
Source: BMC Musculoskelet Disord. 2018 Dec 3;19:431. doi: 10.1186/s12891-018-2351-9 (PMC6278039; doi:10.1186/s12891-018-2351-9)
Supplement: Supplementary file 1 — Table S1. Dropout analysis comparing baseline characteristics of patients included in the analysis of sick leave to those not included. (PDF 63 kb) [file 12891_2018_2351_MOESM1_ESM.pdf]

Additional file 2: Table S2 Dropout analysis comparing baseline characteristics of patients included in the analysis of sick leave to those not included

Variable	Included (n=161)	Number of responders	Missing (n=114)	Number of responders	Difference (p-value)
Sex, female, n (%)	73 (45.34)	161	41 (35.96)	114	0.12
Age, years, mean (SD)	40.07 (10.89)	161	36.72 (10.27)	114	0.01*
Body Mass Index, mean (SD)	25.59 (4.30)	157	26.46 (5.03)	110	0.14
Education after primary school, n (%)		159		109	0.30
<2 years	43 (27.04)		40 (36.70)		
2-4 years	103 (64.78)		64 (58.72)		
>4 years	4 (2.52)		2 (1.83)		
Other	9 (5.66)		3 (2.75)		
Current smoker, no, n (%)	75 (47.17)	159	48 (43.24)	111	0.52
Alcohol, ≤7 units/week, n (%)	120 (77.42)	155	82 (77.36)	106	0.99
Physical activity level leisure, n (%)		157		111	0.08
Little-some	130 (82.80)		95 (85.59)		
Moderate-high	27 (17.20)		16 (14.41)		
Sick leave, yes, n (%)	157 (100)	157		-	-
Duration of sick leave, weeks, mean (SD)	12.15 (17.55)	150		-	-
Employment, no, n (%)	51 (32.48)	157	46 (40.71)	113	0.47
Compensation case, yes, n (%)	30 (19.60)	153	22 (20.56)	107	0.85
Physical job demands, n (%)		146		107	0.05
None	38 (26.02)		23 (21.50)		
Little	21 (14.38)		15 (14.02)		
Some	62 (42.47)		35 (32.71)		
Heavy	25 (17.12)		34 (31.78)		
Physical health, 0-100, mean (SD)	48.49 (7.86)	142	50.03 (8.25)	95	0.15
Mental health, 0-100, mean (SD)	47.93 (10.07)	142	47.89 (10.60)	95	0.98
Depression, 0-4, mean (SD)	1.20 (0.81)	158	1.28 (0.93)	109	0.47
Anxiety, 0-4, mean (SD)	0.72 (0.65)	154	0.82 (0.69)	109	0.24
LBP[1] duration, <12 months, n (%)	93 (61.18)	152	55 (49.44)	111	0.60
Pain intensity, 0-30, mean (SD)	17.81 (6.03)	159	18.99 (4.92)	112	0.08
Age at first episode of LBP[1], years, mean (SD)	30.77 (12.30)	150	26.31 (10.60)	111	<0.01*
Family history of LBP_1, yes, n (%)	72 (45.57)	158	46 (41.07)	112	0.46
Disability, 0-23, mean (SD)	15.00 (4.56)	161	14.08 (4.71)	114	0.11
FAB work[2], 0-42, mean (SD)	29.05 (9.92)	145	30.38 (9.20)	100	0.28
FAB physical activity[3], 0-24, mean (SD)	16.15 (5.34)	149	16.79 (5.32)	100	0.35
Group, intervention, n (%)	92 (57.14)	161	58 (50.88)	114	0.30

SD = standard deviation

*p-value<0.05 indicates significant difference between the two samples

[1]Low back pain

[2]Fear avoidance beliefs about work

[3]Fear avoidance beliefs about physical activity
