# Supplementary material for: Fear avoidance beliefs as a predictor for long-term sick leave, disability and pain in patients with chronic low back pain
Source: BMC Musculoskelet Disord. 2018 Dec 3;19:431. doi: 10.1186/s12891-018-2351-9 (PMC6278039; doi:10.1186/s12891-018-2351-9)
Supplement: Supplementary file 2 — Table S2. Dropout analysis comparing baseline characteristics of patients included in the analysis of disability to those not included. (PDF 62 kb) [file 12891_2018_2351_MOESM2_ESM.pdf]

Additional file 3: Table S3 Dropout analysis comparing baseline characteristics of patients included in the analysis of disability to those not included

Variable	Included (n=369)	Number of responders	Missing (n=190)	Number of responders	Difference (p-value)
Sex, female, n (%)	185 (50.14)	369	78 (41.05)	190	0.04*
Age, years, mean (SD)	40.00 (10.41)	369	36.77 (10.13)	190	<0.01*
Body Mass Index, mean (SD)	25.21 (4.22)	359	26.17 (4.88)	184	0.02*
Education after primary school, n (%)		363		185	<0.01*
<2 years	66 (18.18)		57 (30.81)		
2-4 years	262 (72.18)		108 (58.38)		
>4 years	18 (4.96)		14 (7.57)		
Other	17 (4.68)		6 (3.24)		
Current smoker, no, n (%)	206 (56.44)	365	93 (49.47)	188	0.12
Alcohol, ≤7 units/week, n (%)	273 (75.83)	360	139 (77.22)	180	0.72
Physical activity level leisure, n (%)		362		185	0.23
Little-some	275 (75.97)		149 (80.54)		
Moderate-high	87 (24.03)		36 (19.49)		
Sick leave, yes, n (%)	163 (44.66)	365	105 (56.15)	187	0.01*
Duration of sick leave, weeks, mean (SD)	12.10 (17.26)	157	12.98(13.41)	104	0.64
Employment, no, n (%)	77 (21.21)	363	50 (26.74)	187	0.15
Compensation case, yes, n (%)	55 (15.07)	350	32 (17.58)	182	0.57
Physical job demands, n (%)		360		182	0.06
None	113 (31.39)		50 (27.47)		
Little	55 (15.28)		22 (12.64)		
Some	135 (37.60)		63 (34.62)		
Heavy	57 (15.83)		46 (25.27)		
Physical health, 0-100, mean (SD)	50.60 (8.57)	327	50.99 (8.23)	168	0.62
Mental health, 0-100, mean (SD)	50.02 (10.29)	327	49.71 (10.41)	168	0.75
Depression, 0-4, mean (SD)	1.05 (0.82)	361	1.18 (0.87)	183	0.09
Anxiety, 0-4, mean (SD)	0.65 (0.64)	354	0.74 (0.68)	182	0.14
LBP_1 duration, <12 months, n (%)	187 (53.43)	350	86 (47.51)	181	0.20
Pain intensity, 0-30, mean (SD)	17.42 (5.91)	366	18.13 (5.12)	188	0.14
Age at first episode of LBP[1], years, mean (SD)	28.96 (11.93)	355	25.58 (11.06)	187	<0.01*
Family history of LBP[1], yes, n (%)	162 (44.63)	363	72 (38.92)	185	0.20
Disability, 0-23, mean (SD)	13.81 (4.96)	369	13.63 (4.92)	190	0.68
FAB work[2], 0-42, mean (SD)	23.62 (11.33)	341	25.66 (11.28)	173	0.05
FAB physical activity[3], 0-24, mean (SD)	15.14 (5.38)	346	16.08 (5.22)	176	0.06
Group, intervention, n (%)	210 (56.91)	369	88 (46.32)	190	0.02*

SD = standard deviation

*p-value<0.05 indicates significant difference between the two samples

[1]Low back pain

[2]Fear avoidance beliefs about work

[3]Fear avoidance beliefs about physical activity
