# Supplementary material for: Fear avoidance beliefs as a predictor for long-term sick leave, disability and pain in patients with chronic low back pain
Source: BMC Musculoskelet Disord. 2018 Dec 3;19:431. doi: 10.1186/s12891-018-2351-9 (PMC6278039; doi:10.1186/s12891-018-2351-9)
Supplement: Supplementary file 3 — Table S3. Dropout analysis comparing baseline characteristics of patients included in the analysis of pain to those not included. (PDF 64 kb) [file 12891_2018_2351_MOESM3_ESM.pdf]

Additional file 4: Table S4 Dropout analysis comparing baseline characteristics of patients included in the analysis of pain to those not included

Variable	Included (n=363)	Number of responders	Missing (n=196)	Number of responders	Difference (p-value)
Sex, female, n (%)	181 (49.82)	363	82 (41.84)	196	0.07
Age, years, mean (SD)	39.81 (10.38)	363	37.22 (10.31)	196	<0.01*
Body Mass Index, mean (SD)	25.21 (4.24)	353	26.13 (4.83)	190	0.03*
Education after primary school, n (%)		357		191	0.01*
<2 years	65 (18.21)		58 (30.37)		
2-4 years	258 (72.27)		112 (58.64)		
>4 years	18 (5.04)		14 (7.33)		
Other	16 (4.48)		7 (3.66)		
Current smoker, no, n (%)	154 (45.09)	359	95 (48.87)	194	0.17
Alcohol, ≤7 units/week, n (%)	270 (76.06)	355	142 (78.38)	185	0.98
Physical activity level leisure, n (%)		356		191	0.50
Little-some	269 (75.56)		155 (81.15)		
Moderate-high	87 (24.44)		36 (18.85)		
Sick leave, yes, n (%)	160 (44.57)	359	108 (55.95)	193	0.14
Duration of sick leave, weeks, mean (SD)	12.13 (17.34)	152	12.92 (13.38)	107	0.68
Employment, no, n (%)	76 (21.29)	357	51 (26.42)	193	0.17
Compensation case, yes, n (%)	54 (15.65)	345	33 (17.65)	187	0.55
Physical job demands, n (%)		355		187	0.12
None	111 (31.27)		52 (27.81)		
Little	54 (15.21)		24 (12.83)		
Some	133 (37.44)		65 (34.76)		
Heavy	57 (16.06)		46 (24.60)		
Physical health, 0-100, mean (SD)	50.65 (8.55)	322	50.90 (8.29)	173	0.75
Mental health, 0-100, mean (SD)	50.11 (10.23)	322	49.57 (10.52)	173	0.58
Depression, 0-4, mean (SD)	1.05 (0.82)	357	1.17 (0.87)	188	0.11
Anxiety, 0-4, mean (SD)	0.64 (0.63)	349	0.74 (0.70)	187	0.10
LBP[1] duration, <12 months, n (%)	185 (53.47)	346	88 (47.57)	185	0.20
Pain intensity, 0-30, mean (SD)	18.48 (5.87)	363	18.00 (5.25)	191	0.34
Age at first episode of LBP[1], years, mean (SD)	28.83 (11.82)	349	25.92 (11.39)	193	0.01*
Family history of LBP[1], yes, n (%)	160 (44.81)	357	74 (38.74)	191	0.17
Disability, 0-23, mean (SD)	13.80 (4.97)	363	13.66 (4.89)	196	0.75
FAB work[2], 0-42, mean (SD)	23.64 (11.27)	335	25.56 (11.40)	179	0.07
FAB physical activity[3], 0-24, mean (SD)	15.12 (5.39)	340	16.08 (5.20)	182	0.05
Group, intervention, n (%)	207 (57.02)	363	91 (46.43)	196	0.02*

SD = standard deviation

*p-value<0.05 indicates significant difference between the two samples

[1]Low back pain

[2]Fear avoidance beliefs about work

[3]Fear avoidance beliefs about physical activity
