# Supplementary material for: Fear avoidance beliefs as a predictor for long-term sick leave, disability and pain in patients with chronic low back pain
Source: BMC Musculoskelet Disord. 2018 Dec 3;19:431. doi: 10.1186/s12891-018-2351-9 (PMC6278039; doi:10.1186/s12891-018-2351-9)
Supplement: Supplementary file 4 — Table S4. Simpler model of association between fear avoidance beliefs at baseline and unsuccessful outcome at 12-month follow-up. (PDF 53 kb) [file 12891_2018_2351_MOESM4_ESM.pdf]

Additional file 1: Table S1 Simpler model of the associations between fear avoidance beliefs at baseline and unsuccessful outcome at 12-month follow-up

Variable	Sick leave (n=136)		Disability (n=302)		Pain (n=316)	
	OR	95% CI	OR	95% CI	OR	95% CI
High fear avoidance beliefs about work, 0-42	1.06*	1.01-1.12	1.03*	1.00-1.06	1.04*	1.01-1.06
Smoking						
High pain intensity, 0-30			1.08**	1.02-1.14		
Low pain intensity, 0-30					1.14**	1.08-1.20
Low disability (log), 0-23			1.16**	1.09-1.25		
Duration of low back pain ≥12 months and little physical job demands[#]			3.85*	1.23-12.50		
Male and little physical job demands[#]						

OR = Odds ratio, CI = confidence intervals, *p-value<0.05, **p-value<0.01, [#]interaction

The simpler model of the multiple logistic regression analyses containing only fear avoidance beliefs about work, fear avoidance beliefs about physical activity and the variables found significant in the final model in Table 4
